# TRPP2 is located in the primary cilia of human non-pigmented ciliary epithelial cells

**DOI:** 10.1007/s00417-023-06150-w

**Published:** 2023-06-28

**Authors:** Wenxu Zheng, Focke Ziemssen, Daniela Suesskind, Bogomil Voykov, Sven Schnichels

**Affiliations:** 1 Centre for Ophthalmology, Eberhard Karls University Tübingen, Tübingen, Germany; 2 University Eye Hospital Leipzig, Leipzig, Germany; 3 Klinik und Poliklinik für Augenheilkunde, Liebigstr. 10-14, 72072, Leipzig, Germany

**Keywords:** Intraocular pressure, Mechanosensitive channels, TRPP2, Non-pigmented ciliary epithelial cells, Glaucoma

## Abstract

**Purpose:**

Mechanosensitive channels (MSCs) and primary cilium possess a possible relevance for the sensation of intraocular pressure (IOP). However, there is only limited data on their expression and localization in the ciliary body epithelium (CBE). The purpose of this study was to characterize the expression and localization of TRPP2 in a human non-pigmented ciliary epithelial cell (HNPCE) line.

**Methods:**

The expression of the TRPP2 was studied by quantitative (q)RT-PCR and in situ hybridization in rat and human tissue. Protein expression and distribution were studied by western blot analysis, immunohistochemistry, and immunoelectron microscopy. Cellular location of TRPP2 was determined in rat and human CBE by immunofluorescence and immunoblot analysis. Electron microscopy studies were conducted to evaluate where and with substructure TRPP2 is localized in the HNPCE cell line.

**Results:**

The expression of TRPP2 in rat and human non-pigmented ciliary epithelium was detected. TRPP2 was mainly located in nuclei, but also showed a punctate distribution pattern in the cytoplasm of HNPCE of the tissue and the cell line. In HNPCE cell culture, primary cilia did exhibit different length following serum starvation and hydrostatic pressure. TRPP2 was found to be colocalized with these cilia in HNPCE cells.

**Conclusion:**

The expression of TRPP2 and the primary cilium in the CB may indicate a possible role, such as the sensing of hydrostatic pressure, for the regulation of IOP. Functional studies via patch clamp or pharmacological intervention have yet to clarify the relevance for the physiological situation or aqueous humor regulation.

**Supplementary Information:**

The online version contains supplementary material available at 10.1007/s00417-023-06150-w.


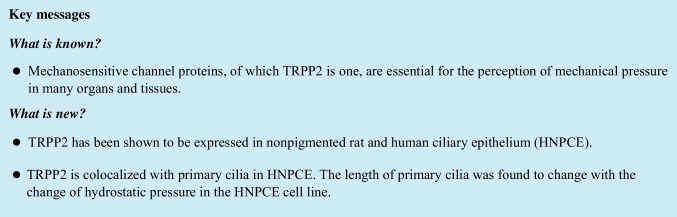


## Introduction

Impaired sensation of intraocular pressure (IOP) contributes to the imbalance between aqueous humor secretion of the ciliary body (CB) and the outflow in the trabecular meshwork (TM). Although little is known on the specific baroreceptors, recent studies describe the potential role of primary cilia in this context. Cilia localized in TM cells are altered in the rare congenital Lowe syndrome with congenital glaucoma [1]. Primary cilia have also been described as potential mechanosensory organelles in cholangiocytes [2], vascular endothelia [3, 4], renal collecting duct epithelium [5], and chondrocytes [6]. A shortening of the primary cilia is observed with increasing hydrostatic pressure; the transient receptor potential vanilloid 4 (TRPV4) is a responsible mechanosensitive channel of the cilia [1]. Therefore, a structured search of other candidates keeps the focus on the transient receptor potential (TRP) cation channel family [7].

Polycystin-2 (TRPP2) has been reported to be located on primary cilia. The channel contributes to fluid-flow sensation in kidney cells [8], vascular endothelial cells [9], and cholangiocytes [2]. The protein is a Ca^2+^ permeable transient receptor potential (TRP) cation channel, whose deficiency may cause autosomal-dominant polycystic kidney disease (ADPKD) [10].

This study was initiated to characterize the ocular expression and localization of TRPP2. The colocalization of TRPP2 with primary cilia was analyzed in human non-pigmented ciliary epithelial (HNPCE) cells, at the site of which the active transport of ions makes an important contribution responsible for the production of aqueous humor in the CB [11]. The carbonate anhydrase is important, controlling local pH with bicarbonate transport through the ciliary epithelium (hydration of CO with formation of HCO and protons). Chloride (Cl) is the major anion transported through the epithelium via Cl channels and, with other actively transported molecules, drives ultrafiltration and diffusion via osmotic gradients. It is still unclear which individual forms of glaucoma might be due to the aforementioned ciliopathies [1]. The aim of these studies was to improve the knowledge of the (ultra)structure in the CB, with focus on TRPP2.

## Materials and methods

### Animal and tissue preparation

Animals were treated according to the Principles of Laboratory Animal Care (NIH publication No. 85–23, revised 1985), the OPRR Public Health Service Policy on the Human Care and Use of Laboratory Animals (revised 1986), and the German animal protection law. Rat eyes of an approved experiment were used (Approval number of the Animal research Committee: HG6/14). Six wildtype female Sprague–Dawley rats (12 weeks old) were sacrificed using carbon dioxide inhalation, and 12 eyes were enucleated immediately after death. After careful dissecting of the whole eyeballs, 3 eyes were fixed for immunohistochemistry, 6 eyes were processed for protein analysis, and 3 eyes were processed for mRNA analysis. For protein and mRNA analysis, the eyes were transferred under a sterile hood and dissected under a microscope into seven parts: the retina, CB, cornea, sclera, optic nerve (ON) iris, and lens. The tissues were subsequently frozen using liquid nitrogen and stored at − 80 °C until further analysis.

### Human ocular specimens

Human eye experiments were conducted according to the tenets of the Declaration of Helsinki, and the study was approved by the local ethics committee (Ethics No.: 332/2021BO2). 3 human eyes that were removed due to choroidal melanoma but with a normal anterior segment were obtained (University of Tübingen, no. 38072, 38,282, 38,375). Only eyes with normal structure of ciliary body were used for analysis.

### HNPCE cell culture

Primary HNPCE cells were purchased from ScienCell Research Laboratories (cat no. 6580, CA, USA). The cells were cultured in epithelial cell medium (ScienCell Research Laboratories, cat no. 4101, CA, USA) at 37 °C in an incubator with 5% CO_2_ and 95% humidity according to the instruction of manufacturer. Originally, the cells were derived from clones obtained from a primary culture of human nonpigmented ciliary epithelium [12].

### Antibodies

Table 1

**Table 1:** List of antibodies used

Primary antibody	Origin	Specificity	Dilution
Rabbit anti-TRPP2	Proteintech (Manchester, UK), 19,126–1-AP	TRPP2	IF (1:200), IHC (1:400), WB (1:300), ICC (1:100)
Jacalin-FITC	EY Laboratories (San Mateo, USA), F-6301–2	NPE cells	IF (1:100)
Mouse anti-acetylated α-tubulin	Sigma-Aldrich (St. Louis, USA), T7451	Primary cilia axoneme	IHC (1:100), ICC (1:200)
Rabbit anti-γ-tubulin	BioLegend (San Diego, USA), 620,901	Primary cilia basal body	ICC (1:200)
Rabbit anti-β-actin	Cell Signaling Technology (Danvers, USA), 4970	β-Actin	WB (1:1000)
Rabbit anti-GAPDH	Cell Signaling Technology (Danvers, USA), 2118	GAPDH	WB (1:1000)
Secondary antibody	Origin	Specificity	Dilution
Alexa Fluor 555-conjugated donkey anti-rabbit IgG	Abcam (Cambridge, UK), ab150074	Rabbit IgG	IF (1:2000)
Alexa Fluor 488-conjugated goat anti-rabbit IgG	Thermo Fisher Scientific (Waltham, USA), A-11008	Rabbit IgG	IF (1:2000)
Alexa Fluor 555-conjugated goat anti-mouse IgG	Thermo Fisher Scientific (Waltham, USA), A-21422	Mouse IgG	IF (1:2000)
IRDye® 800CW goat anti-rabbit IgG	Li-Cor Biosciences Inc. (Lincoln, USA), 926–32,211	Rabbit IgG	WB (1:15,000)

*IF*, immunofluorescence; *IHC*, immunohistochemistry; *WB*, western blot; *ICC*, immunocytochemistry; *NPE*, non-pigmented ciliary epithelium

### Immunofluorescence staining

Horizontal Sects. (12 µm) of the rat tissue (*n* = 6–8/group) were cut with a cryostat by a cryostat (Leica CM3000; Leica, Germany) at -20 °C for immunofluorescence. The slices were fixed with ice-cold methanol for 10 min at room temperature (RT). After washing with Tris-buffered saline (TBS), slices were incubated with Jacalin-FITC, a marker for NPE cell, for 30 min in a dark chamber at RT. Then blocking at RT for 1 h with 5% bovine serum albumin (BSA) in TBS, slices were incubated with the primary antibody TRPP2 (Table 1) at 4 °C overnight. After washing, slices were incubated with the fluorescence-labeled secondary antibody for 1 h at RT. Slides were observed on Axioskop fluorescent microscope (Zeiss, Germany). To ensure avoidance of selection bias for areas of staining, the first field of view of the tissue of interest was selected as start point of analysis. Analysis was performed in a raster fashion to ensure no selection or double counting. Cells were counted as immunopositive or immunonegative, before the percentage of immunopositivity was calculated and compared with the positive control tissue.

### Immunohistochemistry staining

Immunohistochemistry was performed to detect the expression of TRPP2 in human eye tissues. After removing paraffin from human eye, tissue Sects. (4 µm) by using xylol the specimen were rehydrated in a graded alcohol series. Antigen retrieval was performed in citric buffer (pH 6.0). Primary antibody TRPP2 for detection of TRPP2 and mouse anti-acetylated α-tubulin for detection of primary cilia axoneme (Table 1) were incubated at 4 °C overnight. After incubation with secondary antibody, the immunoreaction was detected using a commercially available alkaline phosphatase-anti-alkaline phosphatase kit (Dako REAL™ Detection System, Alkaline phosphatase/red, rabbit/mouse, DakoCytomation GmbH, Hamburg, Germany). Sections were counterstained with Mayer’s hematoxylin (*n* = 4–6/group). For negative controls, sections underwent the same immunohistochemical staining procedure without the primary antibody [13]. The same camera settings (including exposure time) were used for all images. For all analyses, we used an ImageJ macro. After background subtraction, the same lower and upper thresholds were set. The percentage of the labeled areas was measured for each picture using the macro and exported to Excel.

### Western blotting

Tissue and cellular samples were homogenized in cell lysis buffer (cat no. FNN0011, Invitrogen, CA, USA), supplemented with phenylmethylsulfonyl fluoride and a protease inhibitor cocktail (#539,134, Calbiochem, San Diego, USA). After homogenization at 13,000 × g (4 °C) for 10 min, the supernatant was collected for protein analysis. The protein content was determined using a BCA protein assay kit (#23,227, Pierce, Rockford, USA). The same amount of protein was separated on sodium dodecyl sulfate polyacrylamide gel electrophoresis (SDS-PAGE) gels for expression analysis. 10% SDS-PAGE gels (Mini-PROTEAN® TGX™ Gel, #456–1033, Bio-Rad., CA, USA) were used to detect TRPP2. After SDS gel separation, proteins were transferred onto nitrocellulose membranes using a wet transfer technique (350 mA for 1 h). Membranes were blocked for 1 h in 5% nonfat dry milk in TBST. The membranes were incubated with the primary antibody TRPP2 (Table 1) at 4 °C overnight. After washing three times with TBST and incubation with the appropriate secondary antibody for 1 h at RT, membranes (*n* = 3) were detected using an Odyssey Imaging System (Li-Cor Bioscience) [14]. Rat kidney tissue was used as a positive control.

### Serum starvation of HNPCE cells

HNPCE cells were exposed to serum starvation before cilia length was evaluated. Primary HNPCE cell cultures were seeded on poly-L-lysine (PLL)-coated cover slips in 24-well plate at a density of 4.0 × 10^4^ cells/well. On the next day, the growth medium of HNPCE cells was changed from 1% serum medium to no serum medium. After serum starvation for 24, 48, and 72 h, HNPCE cells were used for immunocytochemistry (*n* = 5–6/group).

### Immunocytochemistry staining

After serum-starvation, the HNPCE cell cultured cover slips were treated with 4% paraformaldehyde (PFA) for fixation for 15 min at RT followed by permeabilization with 0.2% Triton X-100. Samples were then blocked with 1% BSA/PBS for 90 min at RT. Primary antibody mouse anti-acetylated α-tubulin and rabbit anti-γ-tubulin (Table 1) for detection of primary cilia axoneme and basal body, rabbit anti-TRPP2, and mouse anti-acetylated α-tubulin (Table 1) for colocalization of TRPP2 and primary cilia were applied at 4 °C overnight, followed by secondary antibodies for 1 h at RT. Slides (*n* = 5–6/group) were observed on fluorescent microscope (Zeiss Axioskop). Imaging was analyzed with an Axio (Version 4.8) for cilia measurements.

### Total RNA extraction and reverse transcription PCR

mRNA was extracted from rat eye tissue (*n* = 3–6/group) or HNPCE cells (*n* = 6–8/group) and directly synthesized to cDNA using the Milteny MultiMACS cDNA Synthesis Kits according to the manufacturer’s instructions. Synthesized cDNA was stored at -20℃ for further analysis. Primers were designed for *TRPP2* by using NCBI’s Primer-BLAST. The sequences for human *TRPP2* were forward-TCGACAAGATCTCAAAGGGA and reverse-GTCTCACCAGGACTTGAAAC; for rat were forward-AGGACCTAGACTTGGAACAC and reverse-GTGGATCTCACTATCCCGAC. Conventional PCR was performed with Go Taq® Hot Start Polymerase (M5001, Promega, US) in the UNO Cycler PCR system (VWR, UK). 2 μl of undiluted cDNA was used to amplify the *TRPP2* sequences in a 25-μl reaction volume. The thermocycling conditions were 5 min at 94 °C, followed by 45 cycles of 30 s at 94 °C, 30 s at 58 °C, and 30 s at 72 °C. The final elongation step was 7 min at 72 °C. After conventional PCR, the amplicons were resolved on 1.0% agarose gels for electrophoresis. Gels were then detected using the Odyssey Imaging System (Li-Cor Bioscience).

### Electron microscopy

The HNPCE cell was fixed overnight at 4 °C in 2% glutaraldehyde in 0.1 M cacodylate buffer (pH 7.4) and post fixed with 1% OsO4% at room temperature in 0.1 M cacodylate buffer for 1 h, stained with uranyl acetate, and embedded in Epon after dehydration in a graded series of ethanol. Ultrathin sections were made and analyzed with a Zeiss 900 electron microscope (Zeiss, Jena, Germany) [15].

### Statistical analyses

Regarding immunohistology and western blot, data are presented as the mean ± standard error of the means (SEMs), unless otherwise noted. Statistical comparisons were calculated by unpaired Student’s t test using SPSS Statistics (Version 26.0) software (IBM, Armonk, NY, USA). A *p* value ≤ 0.05 was considered as statistically significant.

## Results

### Expression of TRPP2 in rat eyes

**Fig. 1:**
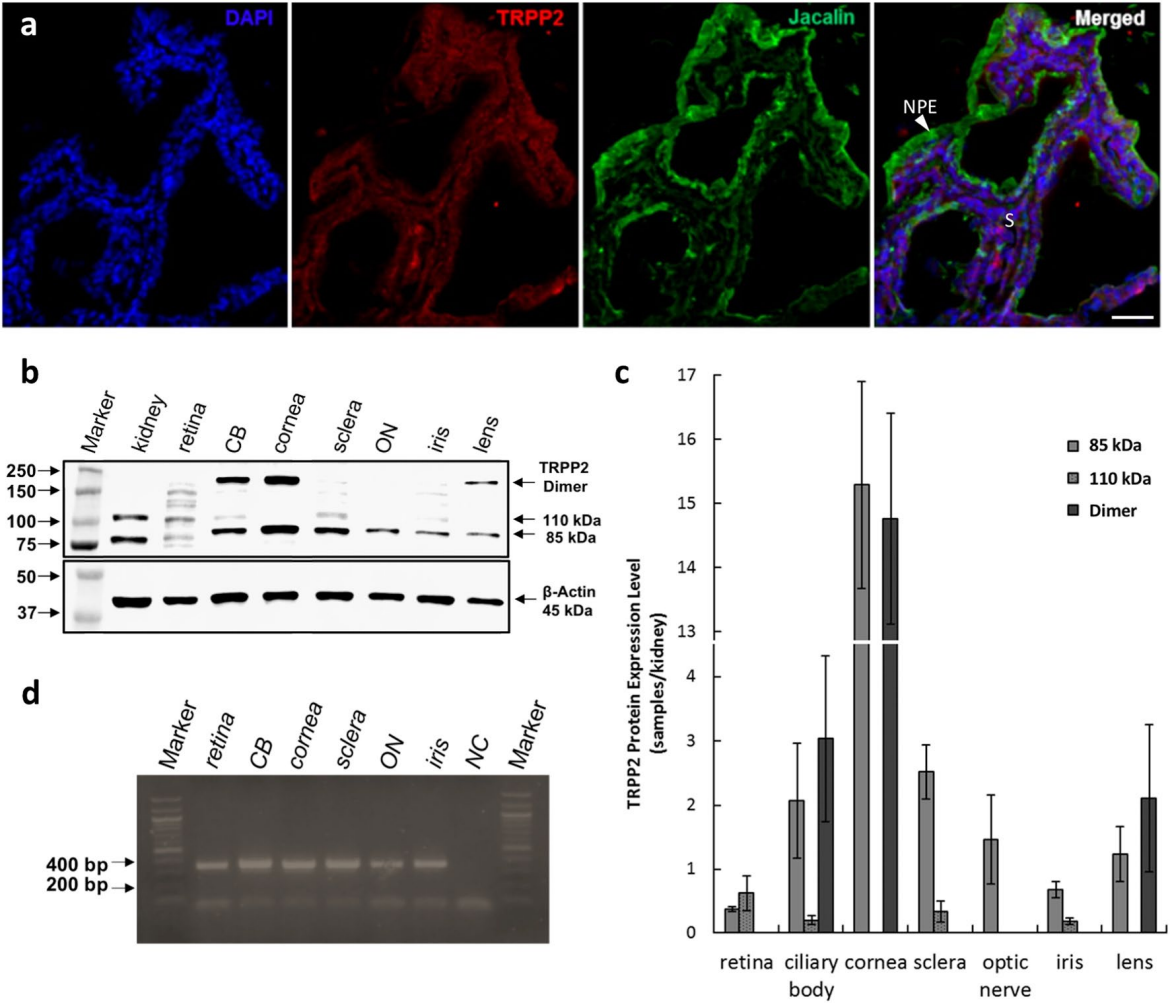
Localization of TRPP2 in rat eye tissue (*n* = 6). **a** TRPP2 was distributed in the rat ciliary body. TRPP2 antibody (red); Jacalin-FITC (green); DAPI (blue). Scale bar: 50 µm (*n* > 20 sections). NPE, non-pigmented epithelium; S, stroma. **b** Western blot analysis showed the protein expression level of TRPP2 in different tissues of a rat eye (*n* = 3). The largest band (220 kDa) is the dimer of TRPP2, the 110 kDa and 85 kDa bands represent two different isoforms of TRPP2. **c** Quantitative analysis of three different forms of TRPP2 protein in different tissue. The expression of TRPP2 was normalized to β-actin. Three times of replications were done. **d** Reverse transcription PCR showed the mRNA expression level of TRPP2 in different tissues of rat eye (*n* = 6). A 344 bp band represents the PCR product of TRPP2. CB, ciliary body; ON, optic nerve; NC, negative control

Positive TRPP2 staining in rat ciliary body (CB) was shown via immunofluorescence. Jacalin confirmed the localization in NPE cells of the ciliary body. Protein expression analysis revealed TRPP2 abundance in the non-pigmented epithelium (NPE), pigmented epithelium (PE), and stroma of CB (Fig. 1a).

Western blot analysis was performed to study the expression level of TRPP2 in different structures of the rat eye. Three different forms of TRPP2 protein were observed by western blot. The largest band (220 kDa) is the dimer of TRPP2, while the 110 kDa and 85 kDa bands represent two different isoforms of TRPP2. The dimer of TRPP2 was highly expressed in the cornea (14.76 ± 1.65), CB (3.03 ± 1.30), and the lens (2.10 ± 1.15), but not in other parts. The isoform with 110 kDa was expressed weakly in the retina (0.62 ± 0.27), sclera (0.32 ± 0.16), CB (0.19 ± 0.06), and iris (0.18 ± 0.05), but not in other tissues. The 85 kDa isoform was highly expressed in the CB (2.06 ± 0.90), cornea (15.28 ± 1.61), sclera (2.51 ± 0.42), ON (1.46 ± 0.70), and lens (1.23 ± 0.43) but weakly expressed in the retina (0.37 ± 0.04) and iris (0.67 ± 0.13) (Fig. 1b and c).

Additional analysis with reverse transcription PCR confirmed expression of TRPP2 in different tissues of the rat eye. A 344 bp band represented the PCR product of TRPP2 (Fig. 1d), further sanger sequencing validated the PCR product (Supplementary Material, Fig. S1).

### Expression of TRPP2 in human ciliary body and HNPCE cells

**Fig. 2:**
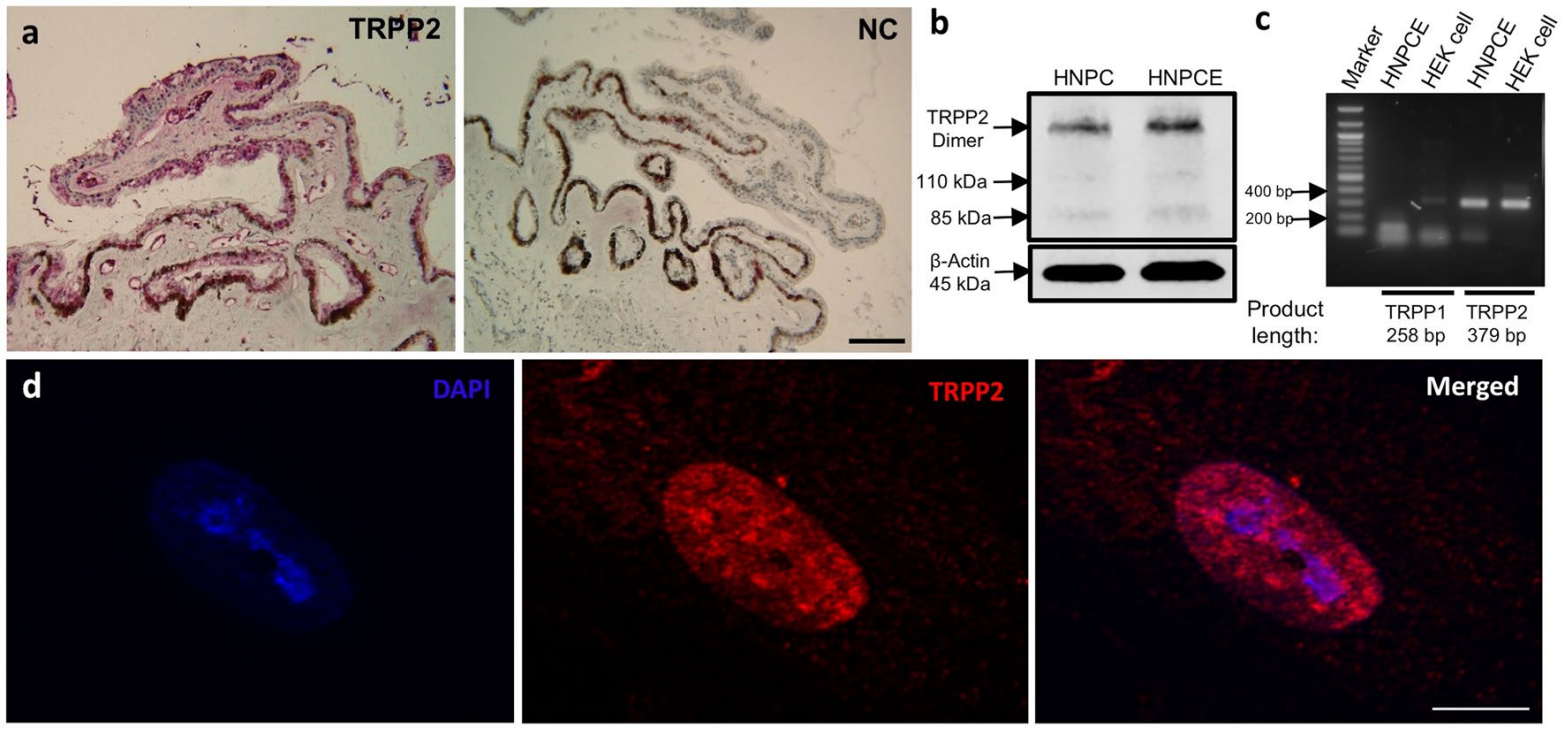
The expression of TRPP2 in human ciliary body and HNPCE cells. **a** TRPP2 is expressed in the human ciliary body epithelium (*n* = 6). TRPP2 antibody (red); nuclei (blue). Scale bar: 50 µm (*n* = 3). **b** Western blot analysis showed the expression of TRPP2 in HNPCE cells; β-actin was used as loading control (*n* = 3). **c** TRPP2 mRNA expression was detected by reverse transcript PCR. **d** Immunofluorescence staining displays that the TRPP2 (red) localized in nuclei (via DAPI staining; blue) and partly in cytoplasm of HNPCE cells. Scale bar: 10 µm

TRPP2 staining was analyzed in human tissue. Similarly to the rats, we observed the expression of TRPP2 in non-pigmented as well as pigmented cells of the human CB (Fig. 2a). Furthermore, the expression of TRPP2 in HNPCE cell culture was tested by using western blot analysis (Fig. 2b), PCR (Fig. 2c), and immunostaining (Fig. 2d). In agreement with the result of immunohistochemistry staining of the human ciliary body, immunostaining, PCR, and western blot results all proved the expression of TRPP2 in HNPCE. Interestingly, we observed that TRPP2 was mainly located in nuclei and in punctate distribution in the cytoplasm of HNPCE cells (Fig. 2d).

### Serum starvation induced the formation of primary cilia in HNPCE cells

**Fig. 3:**
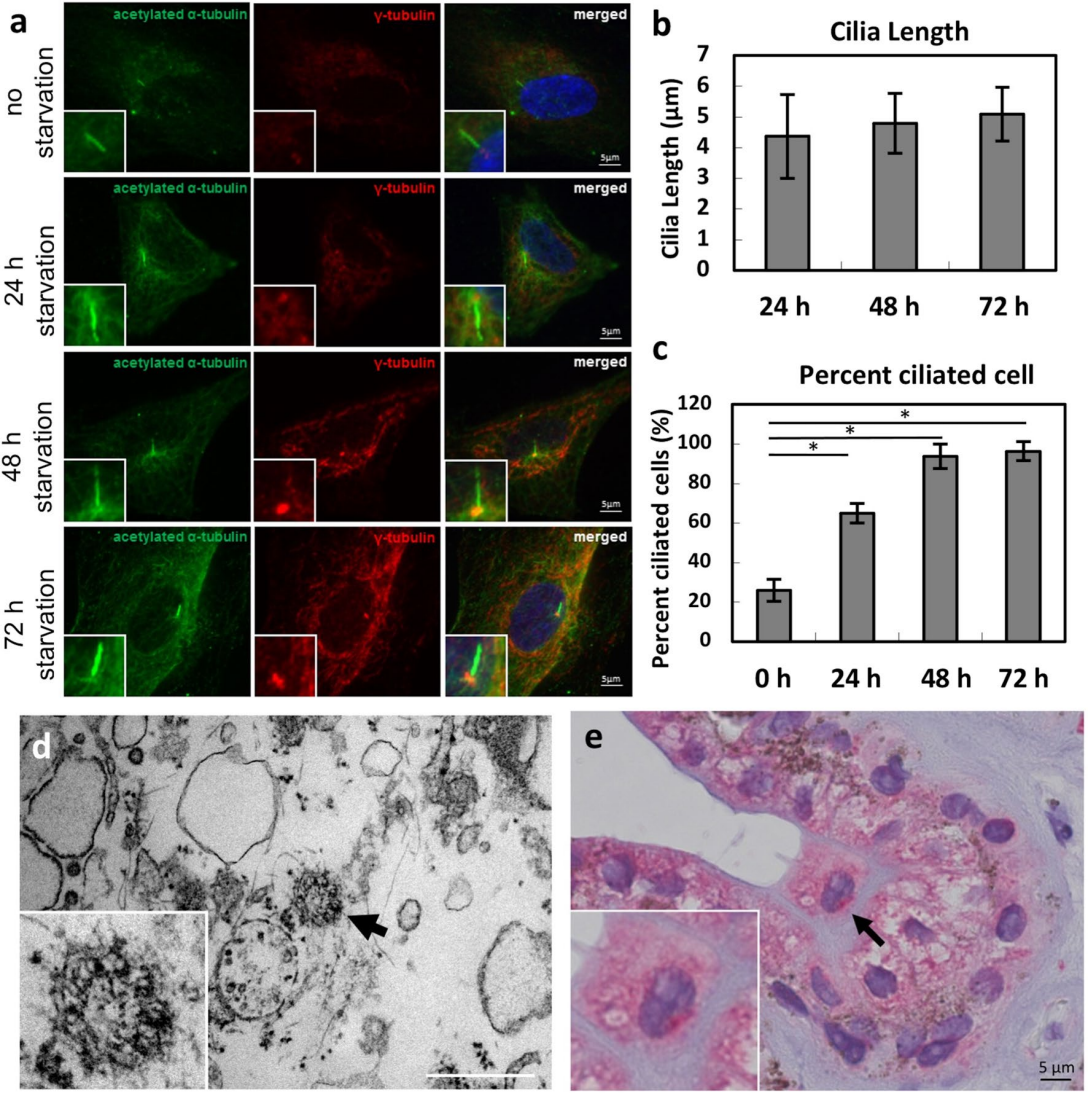
Formation of primary cilia in HNPCE cell culture and human ciliary body epithelium. **a** Serum starvation of HNPCE cells was performed at 0, 24, 48, and 72 h (*n* = 6). Representative immunofluorescence photomicrographs showing cilia formation with anti-acetylated α-tubulin (green) antibody and γ-tubulin (red) antibody (DAPI, blue). Cilia length (**b**) and ciliation rate (**c**) are given. Error bars represent SD (SD). *n* > 50 cilia, three independent experiments, ANOVA, ^*^p < 0.05. (Scale bar, 5 μm). An increase of cilia from 26.0 ± 5.6% to 96.4 ± 4.7% was seen after 72 h, accompanied only by a slight elongation of 4.4 µm ± 1.0 μm to 5.1 µm ± 0.9 µm. **d** Electron micrograph showed a primary cilium in HNPCE. Arrow shows the crossing section of primary cilium. (Scale bar, 500 nm). **e** Immunohistochemical staining detected a primary cilium in HNPCE of the human eye. Arrow shows primary cilium. (Scale bar, 5 μm)

The expression of TRPP2 and structure of primary cilia were examined more closely: acetylated α-tubulin and rabbit anti-γ-tubulin for detection of primary cilia axoneme and basal body were observed in cultured HNPCE cells by immunofluorescence staining (Fig. 3a). First, the length of primary cilia was calculated at different time points after serum starvation directly on the AxioVision Viewer (AxioVs40 V 4.8.2.0; Carl Zeiss MicroImaging, Germany). Extension of the starvation time to 24 h, 48 h, and 72 h, respectively, did not affect the length of primary cilia. After 24 h of serum starvation, HNPCE cells formed 4.4 μm (4.4 ± 1.4 μm) primary cilia in length. After 48 h of starvation, the length of primary cilia remained almost the same (4.8 ± 1.0 μm). After 72 h of starvation, the average length of the primary cilia was 5.1 μm (5.1 ± 0.9 μm). No significant difference was observed between different time points (Fig. 3b). Next, the cells with primary cilia were counted at different time points of serum starvation. Interestingly, our results showed that extension of the time of serum starvation could induce the formation of primary cilia in HNPCE cells. Without serum starvation (at 0 h) only 26.0% (± 5.6%) of cells formed primary cilia. After serum starvation for 24 h, the percentage of cells with primary cilia increased to 65% (± 4.9%). Extended serum starvation time to 48 or 72 h caused almost all HNPCE cells to form primary cilia (93.9 ± 6.1% at 48 h and 96.4 ± 4.7% at 72 h) (Fig. 3c).

### Primary cilia formation in HNPCE cells of human ciliary body

In HNPCE cell culture, the typical sign of primary cilia, a “9 + 0” arrangement was observed and confirmed (Fig. 3d). The electron microscopic images verified that HNPCE cells can form primary cilia. Immunohistochemistry staining of cilia structure was performed in human ciliary body epithelium, and we observed primary cilia in the non-pigmented epithelium of the ciliary body (Fig. 3e). Thus, the existence of primary cilia was approved in the HNPCE cells in vitro and in vivo.

### Colocalization of primary cilia and TRPP2

**Fig. 4:**
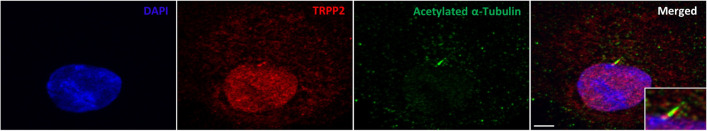
Immunostaining for TRPP2 co-localized to primary cilia in HNPCE cells (*n* = 5). Double immunostaining was performed on HNPCE cells after serum-starved for 48 h. TRPP2 antibody (red); acetylated α-tubulin (green); DAPI (blue). (Scale bar, 5 μm)

The co-localization of TRPP2 and acetylated α-tubulin, primary cilia marker, was examined by using double immunofluorescence staining. TRPP2 was distributed on the axoneme and the basal body of primary cilia in HNPCE cells with 48 h serum-starvation (Fig. 4). Although no functional studies as a function of TRPP2 have yet taken place, previous observations regarding cilia length as a function of hydrostatic pressure have been confirmed.

## Discussion

The expression of the mechano-sensitive membrane protein TRPP2 within the CB epithelium was confirmed, in rat (Fig. 1) as well as in human eyes (Fig. 2). The detection was not limited to non-pigmented cells. We found a co-localization of TRPP2 and primary cilia, very similar to the situation as it was pre-described in the trabecular meshwork for TRPV4 [1].

Earlier reports also indicate the role of TRPP2 in sensing mechanical pressure. For example, the polycystin complex, which is assembled by TRPP2 and its molecular chaperones of polycystin-1, can be activated and induced Ca^2+^ influx to primary by mechanical pressure on several stress-bearing cells, like kidney cells [8], vascular endothelial cells [9], vascular smooth muscle cells [16], and bronchial smooth muscle cells [17]. A case report showed that a polycystic kidney disease (PKD) patient had experienced rapid loss of vision during the terminal stages of his illness, and the postmortem examination revealed glaucomatous changes in both eyes [18]. Thus, there might be a potential connection between glaucoma and polycystic kidney disease secondary to TRPP2.

So far, in eye tissue, TRPP2 has been identified in human TM tissue and primary TM tissue by using immunohisto- and immunocytochemistry staining and western blot and mass spectrometric analyses [19]. Obermüller et al. showed that TRPP2 was punctuated distribution in cytoplasmic of rat corneal epithelium [20]. In mouse retina, TRPP2 expression was also detected through RT-PCR, northern blot, in situ hybridization, and immunodetection [21]. In the retina of a normal SD rat, the endogenous polycystin-2 (TRPP2) was present in the ganglion cell layer, inner nuclear layer (INL), outer nuclear layer (ONL), and photoreceptor cells [22]. Choi et al. using three methods of PCR identified that TRPP2 was expressed in the mouse optic nerve head [23]. Our study for the first time showed the distribution of TRPP2 in both rat and human CB, as well as in HNPCE cells. The formation of aqueous humor is attributed to the ciliary epithelium of CB [11].

Although their significance for certain forms of glaucoma are uncertain or questionable, primary cilia have been described as mechanosensory organelles in a wide range of other cells [2–6]. The primary cilium membrane is constituted by a microtubule based axoneme and anchored through a basal body [24]. In this study, we observed the primary cilia in a cell line of HNPCE cells and human ciliary body by using immunostaining of acetylated α-tubulin also known as Arl13b, a small GTPase which localized in the cilia. The basal body of primary cilia was detected by using γ-tubulin staining. After immunofluorescence, co-staining of TRPP2, and primary cilia, we observed the colocalization of TRPP2 and primary cilia (Fig. 4), which may support that during the IOP regulation, TRPP2 may sense the IOP or at least participate in this procedure.

Previous reports showed that TRPP2 on primary cilia may function not only as a mechanosensitive channel [25] but also as a regulator of other mechanosensitive channels, because TRPP2 can be assembled as homo-tetramer [26] and hetero-tetramer with polycystin-1 (PKD1) [27], TRPC1 [26], and TRPV4 [28]. TRPP2 interacts with popycystin-1(PKD1) to form a 3:1 complex channel by C-terminal coiled-coil domain [27]. TRPP2 and TRPC1 form a hetero-tetramer with a 2:2 subunit and an alternating arrangement [26]. Similarly, as TRPP2 and TRPC1, TRPP2 and TRPV4 also form a hetero tetramer with a 2:2 alternating subunit arrangement [28]. Hetero-tetramer also could be assembled by three different kinds of TRP subfamily; Du et al. found TRPV4, TRPC1, and TRPP2 can assemble to form a heteromeric TRPV4-C1-P2 complex heteromeric channel. The fourth subunit in this tetrameric channel could be anyone of the three channels [29]. Recently, a hetero-multimer included both TRPM3 and TRPP2 subunits were also found that might exist in primary cilia of renal epithelia cells by Kleene et al. [30]. TRPP2 can not only form a hetero tetramer with other TRP family members but could also interact with other MSCs. For example, TRPP2 may inhibit PIEZO1-dependent stretch-activated channels in renal tubular epithelial cells [31]. TRPP2 can also block the mechanical activation of TREK1 by a filamin A-mediated cytoskeletal mechanism [32]. Interestingly, TRPP2 could be inhibited by high levels of PI(4,5)P2 in the membrane [33], while lack of mutation of OCRL lead to accumulation of PI(4,5)P2. The OCRL, a phosphoinositide 5-phosphatases, is well known to metabolize PI(4,5)P2 into PI(4)P. Mutation of OCRL can cause Lowe syndrome, which includes congenital cataract and glaucoma [34]. Thus, these data indicate that TRPP2 may play an important role in regulating mechanosensitive processes through OCRL [1]. The detection of TRPP2 in the HNPCE cell line should not be confused with the proof of the relevance in human TM cells. Furthermore, the presence of TRPP2 on primary cilia does not necessarily mean that they play a role in regulating IOP in the eye. This evidence remains to be verified by functional/electrophysiological methods.

In the present study, we investigated whether serum starvation affected the formation of primary cilia. Serum starvation is a widely described method of inducing primary cilia [35]. Using this method, we observed that the average lengths of primary cilia did not change significantly when the starvation time is extended from 24 to 72 h. This result is different from Luo’s study in primary human trabecular meshwork (TM) cells in which the average lengths of cilia are increased followed serum deprivation [1]. It is unclear whether these differences are due to the fact that the HNPCE cells are different from TM or primary human cells. Similarly to the TM cells, increasing serum starvation time leads to increased percentage of cells with primary cilia. In TM cells, primary cilia are critical for response to pressure changes, and the primary cilia in TM cells can be shortened in response to fluid flow or elevated hydrostatic pressure [1]. Whether the length of primary cilia in human tissue will change following high pressure needs to be investigated.

We acknowledge that there are some important limitations of this study. The direct connection of TRPP2 and primary cilia to IOP regulation needs further characterization. No causal mutation of TRPP2 has been characterized yet, affecting the function of the ciliary body. The non-selective cation-channel TRPP2 is expressed in various tissues including epithelial cells, vascular smooth muscle cells, endothelial cells, cardiac myocytes, adrenal glands, and ovaries [9, 16, 17, 36]. By forming homomultimeric or heteromultimeric channels with the same or other subfamilies, the function depends on the subcellular distribution [37]. In vascular smooth muscle cells, TRPP2 is primarily located in the sarcoplasmic reticulum (SR) and differentially regulates the myogenic response in different vascular beds [16]. The application of TRPP2-specific siRNA, leading to the knockdown of TRPP2, resulted in a reduction of swelling-induced cation currents in vascular smooth muscle cells and a decrease in pressure-induced constriction in cerebral arteries (resistance size) [38]. In endothelial cells, the role as a mechanosensing Ca^2+^ channel conveys a flow-induced vascular dilation [39]. Decreased TRPP2 expression was found to be associated with abnormal vascular function and decreased NO levels. In high salt intake-induced hypertensive rats, TRPP2 protein expression was increased in the vascular smooth muscle cells of the thoracic aorta and mesenteric arteries [40].

The breadth and different role in the response to mechanical signals, from shear stress to blood pressure, is explained by the interaction with different molecules [41]. In reconstituted lipid bilayers, the amplitude and frequency of ionic oscillations of TRPP2 were strongly dependent on the applied voltage, the Ca^2+^ gradient, and the presence of high Ca^2+^ [42].

In summary, our data demonstrate the expression of TRPP2 in the ciliary body and the colocalization of TRPP2 with primary cilia, suggesting useful/necessary experiments to test the possible role of TRPP2 in the sensation of IOP. Although the importance in the regulation of hydrostatic pressure within the posterior chamber cannot yet be assessed, the involvement of TRPP2 in the complex control seems conceivable.

### Supplementary Information

Below is the link to the electronic supplementary material.

Supplementary file1. Fig. S1 Sanger sequencing confirmed the PCR product of TRPP2 (JPG 989 KB)

## Data Availability

The data used to support this study are available from the corresponding author upon reasonable request.

## References

[1] Luo N, Conwell MD, Chen X, Kettenhofen CI, Westlake CJ, Cantor LB, Wells CD, Weinreb RN, Corson TW, Spandau DF, Joos KM, Iomini C, Obukhov AG, Sun Y (2014) Primary cilia signaling mediates intraocular pressure sensation. Proc Natl Acad Sci USA 111:12871-12876 10.1073/pnas.1323292111 PMC4156748 25143588

[2] Masyuk AI, Masyuk TV, Splinter PL, Huang BQ, Stroope AJ, LaRusso NF (2006). Cholangiocyte cilia detect changes in luminal fluid flow and transmit them into intracellular Ca2+ and cAMP signaling. Gastroenterology.

[3] Iomini C, Tejada K, Mo W, Vaananen H, Piperno G (2004). Primary cilia of human endothelial cells disassemble under laminar shear stress. J Cell Biol.

[4] Nauli SM, Kawanabe Y, Kaminski JJ, Pearce WJ, Ingber DE, Zhou J (2008). Endothelial cilia are fluid shear sensors that regulate calcium signaling and nitric oxide production through polycystin-1. Circulation.

[5] Liu X, Vien T, Duan J, Sheu SH, DeCaen PG, Clapham DE (2018). Polycystin-2 is an essential ion channel subunit in the primary cilium of the renal collecting duct epithelium. Elife.

[6] Wann AK, Zuo N, Haycraft CJ, Jensen CG, Poole CA, McGlashan SR, Knight MM (2012). Primary cilia mediate mechanotransduction through control of ATP-induced Ca2+ signaling in compressed chondrocytes. Faseb j.

[7] Lin SY, Corey DP (2005). TRP channels in mechanosensation. Curr Opin Neurobiol.

[8] Nauli SM, Alenghat FJ, Luo Y, Williams E, Vassilev P, Li X, Elia AE, Lu W, Brown EM, Quinn SJ, Ingber DE, Zhou J (2003). Polycystins 1 and 2 mediate mechanosensation in the primary cilium of kidney cells. Nat Genet.

[9] AbouAlaiwi WA, Takahashi M, Mell BR, Jones TJ, Ratnam S, Kolb RJ, Nauli SM (2009). Ciliary polycystin-2 is a mechanosensitive calcium channel involved in nitric oxide signaling cascades. Circ Res.

[10] Mochizuki T, Wu G, Hayashi T, Xenophontos SL, Veldhuisen B, Saris JJ, Reynolds DM, Cai Y, Gabow PA, Pierides A, Kimberling WJ, Breuning MH, Deltas CC, Peters DJ, Somlo S (1996). PKD2, a gene for polycystic kidney disease that encodes an integral membrane protein. Science.

[11] Civan MM, Macknight AD (2004). The ins and outs of aqueous humour secretion. Exp Eye Res.

[12] Martin-Vasallo P, Ghosh S, Coca-Prados M (1989). Expression of Na, K-ATPase alpha subunit isoforms in the human ciliary body and cultured ciliary epithelial cells. J Cell Physiol.

[13] Suesskind D, Schatz A, Schnichels S, Coupland SE, Lake SL, Wissinger B, Bartz-Schmidt KU, Henke-Fahle S (2012). GDF-15: a novel serum marker for metastases in uveal melanoma patients. Graefes Arch Clin Exp Ophthalmol.

[14] Castellani L, Root-Mccaig J, Frendo-Cumbo S, Beaudoin MS (1985). Wright DC (2014) Exercise training protects against an acute inflammatory insult in mouse epididymal adipose tissue. J Appl Physiol.

[15] Tschulakow AV, Oltrup T, Bende T, Schmelzle S, Schraermeyer U (2018). The anatomy of the foveola reinvestigated. Peer J.

[16] Lu CJ, Du H, Wu J, Jansen DA, Jordan KL, Xu N, Sieck GC, Qian Q (2008). Non-random distribution and sensory functions of primary cilia in vascular smooth muscle cells. Kidney Blood Press Res.

[17] Wu J, Du H, Wang X, Mei C, Sieck GC, Qian Q (2009). Characterization of primary cilia in human airway smooth muscle cells. Chest.

[18] Berkley WL (1951). Glaucoma associated with polycystic kidney disease. Am J Ophthalmol.

[19] Tran VT, Ho PT, Cabrera L, Torres JE, Bhattacharya SK (2014). Mechanotransduction channels of the trabecular meshwork. Curr Eye Res.

[20] Obermüller N, Gallagher AR, Cai Y, Gassler N, Gretz N, Somlo S, Witzgall R (1999). The rat pkd2 protein assumes distinct subcellular distributions in different organs. Am J Physiol.

[21] Gilliam JC, Wensel TG (2011). TRP channel gene expression in the mouse retina. Vision Res.

[22] Gallagher AR, Hoffmann S, Brown N, Cedzich A, Meruvu S, Podlich D, Feng Y, Könecke V, de Vries U, Hammes HP, Gretz N, Witzgall R (2006). A truncated polycystin-2 protein causes polycystic kidney disease and retinal degeneration in transgenic rats. J Am Soc Nephrol.

[23] Choi HJ, Sun D, Jakobs TC (2015). Astrocytes in the optic nerve head express putative mechanosensitive channels. Mol Vis.

[24] Ishikawa H, Marshall WF (2011). Ciliogenesis: building the cell's antenna. Nat Rev Mol Cell Biol.

[25] Scarinci N, Perez PL, Cantiello HF, Cantero MDR (2022). Polycystin-2 (TRPP2) regulates primary cilium length in LLC-PK1 renal epithelial cells. Front Physiol.

[26] Kobori T, Smith GD, Sandford R, Edwardson JM (2009). The transient receptor potential channels TRPP2 and TRPC1 form a heterotetramer with a 2:2 stoichiometry and an alternating subunit arrangement. J Biol Chem.

[27] Zhu J, Yu Y, Ulbrich MH, Li MH, Isacoff EY, Honig B, Yang J (2011) Structural model of the TRPP2/PKD1 C-terminal coiled-coil complex produced by a combined computational and experimental approach. Proc Natl Acad Sci USA 108:10133-10138 10.1073/pnas.1017669108 PMC3121833 21642537

[28] Stewart AP, Smith GD, Sandford RN, Edwardson JM (2010). Atomic force microscopy reveals the alternating subunit arrangement of the TRPP2-TRPV4 heterotetramer. Biophys J.

[29] Du J, Ma X, Shen B, Huang Y, Birnbaumer L, Yao X (2014). TRPV4, TRPC1, and TRPP2 assemble to form a flow-sensitive heteromeric channel. Faseb j.

[30] Kleene SJ, Siroky BJ, Landero-Figueroa JA, Dixon BP, Pachciarz NW, Lu L, Kleene NK (2019). The TRPP2-dependent channel of renal primary cilia also requires TRPM3. PLoS One.

[31] Peyronnet R, Martins JR, Duprat F, Demolombe S, Arhatte M, Jodar M, Tauc M, Duranton C, Paulais M, Teulon J, Honore E, Patel A (2013). Piezo1-dependent stretch-activated channels are inhibited by polycystin-2 in renal tubular epithelial cells. EMBO Rep.

[32] Li Fraine S, Patel A, Duprat F, Sharif-Naeini R (2017). Dynamic regulation of TREK1 gating by polycystin 2 via a filamin A-mediated cytoskeletal mechanism. Sci Rep.

[33] Ma R, Li WP, Rundle D, Kong J, Akbarali HI, Tsiokas L (2005). PKD2 functions as an epidermal growth factor-activated plasma membrane channel. Mol Cell Biol.

[34] Luo N, West CC, Murga-Zamalloa CA, Sun L, Anderson RM, Wells CD, Weinreb RN, Travers JB, Khanna H, Sun Y (2012). OCRL localizes to the primary cilium: a new role for cilia in Lowe syndrome. Hum Mol Genet.

[35] Nachury MV, Loktev AV, Zhang Q, Westlake CJ, Peränen J, Merdes A, Slusarski DC, Scheller RH, Bazan JF, Sheffield VC, Jackson PK (2007). A core complex of BBS proteins cooperates with the GTPase Rab8 to promote ciliary membrane biogenesis. Cell.

[36] Ong AC (2000). Polycystin expression in the kidney and other tissues: complexity, consensus and controversy. Exp Nephrol.

[37] Tian PF, Sun MM, Hu XY, Du J, He W (2022). TRPP2 ion channels: the roles in various subcellular locations. Biochimie.

[38] Narayanan D, Bulley S, Leo MD (2013). Smooth muscle cell transient receptor potential polycystin-2 (TRPP2) channels contribute to the myogenic response in cerebral arteries. J Physiol.

[39] Hisatsune C, Kuroda Y, Nakamura K (2004). Regulation of TRPC6 channel activity by tyrosine phosphorylation. J Biol Chem.

[40] Zhao R, Zhou M, Li J (2015). Increased TRPP2 expression in vascular smooth muscle cells from high-salt intake hypertensive rats: the crucial role in vascular dysfunction. Mol Nutr Food Res.

[41] Du J, Fu J, Xia XM, Shen B (2016). The functions of TRPP2 in the vascular system. Acta Pharmacol Sin.

[42] Velázquez IF, Cantiello HF, Cantero MDR (2023). High calcium transport by polycystin-2 (TRPP2) induces channel clustering and oscillatory currents. Biochem Biophys Res Commun.

