# Supplementary figures and images for: TRPP2 is located in the primary cilia of human non-pigmented ciliary epithelial cells
Source: Graefes Arch Clin Exp Ophthalmol. 2023 Jun 28;262(1):93–102. doi: 10.1007/s00417-023-06150-w (PMC10806040; doi:10.1007/s00417-023-06150-w)

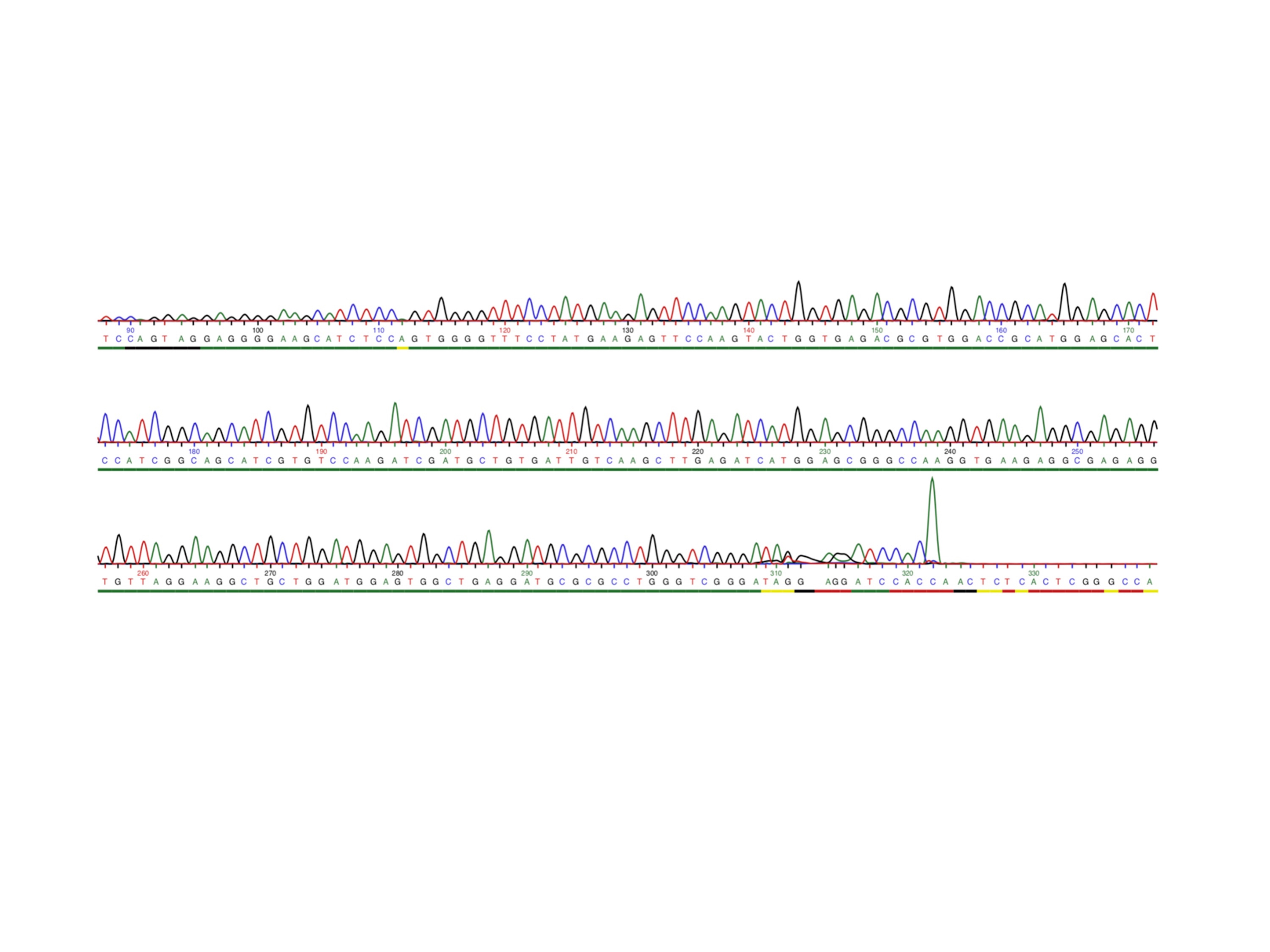

Supplement: Supplementary file 1 — Supplementary file1. Fig. S1 Sanger sequencing confirmed the PCR product of TRPP2 (JPG 989 KB) [file 417_2023_6150_MOESM1_ESM.jpg]
